# Supplementary material for: The Small Ras Superfamily GTPase Rho4 of the Maize Anthracnose Fungus Colletotrichum graminicola Is Required for β-1,3-glucan Synthesis, Cell Wall Integrity, and Full Virulence
Source: J Fungi (Basel). 2022 Sep 22;8(10):997. doi: 10.3390/jof8100997 (PMC9604917; doi:10.3390/jof8100997)
Supplement: Supplementary file 1 [file jof-08-00997-s001.zip › jof-1908711-supplementary.pdf]

Journal of Fungi

Oliveira-Garcia et al. (2022)

Supplementary Materials:

Table S1. PCR primers used in this study.

Nourse1pNR1-fw	5′-TGACCGGTGCCTGGATCTTCCTATAGAATC-3′
Nourse2pNR1-rv	5′-GGTCGGCATCTACTCTATTCCTTTGCCCTC-3′
CgPrho4-fw	5′-GCCAGTGGAATTCTGATTGC-3′
CgPrho4_5'-flank-rv	5′-GAATGCGGCTCTAGAGCGGCGGCAGACTCTAAGTGGTGTG -3′
CgTrho4_3'-flank-fw	5′-CTTAATGATGATTACTAACAGATATCAAGCTTAACTTCAGTCACCGCTCCTC -3′
CgTrho4-rv	5′-ACCCAATGCTGCTACAGGAG-3′
CgPrho4nest-fw	5′-GATGGAAGCGTTCCTTCTTG-3′
CgTrho4nest-rv	5′-CCCGAGTGTCTAGGTATGGTACG-3′
CgRho4probe-fw	5′-TTTACACTTGCCCTCCTTGC-3′
CgRho4probe-rv	5′-CGGCAGACTCTAAGTGGTGTG-3′
CgRho4-RT-PCR-fw	5′-GGCGACGGTGGTTGCG-3′
CgRho4-RT-PCR-rv	5′-CCAGACGGCGGGTGTGT-3′
Cg_ITS2-F1.1	5′-CGTCGTAGGCCCTTAAAGGTAG-3′
Cg_ITS2-R1	5′-TTACGGCAAGAGTCCCTC-3′

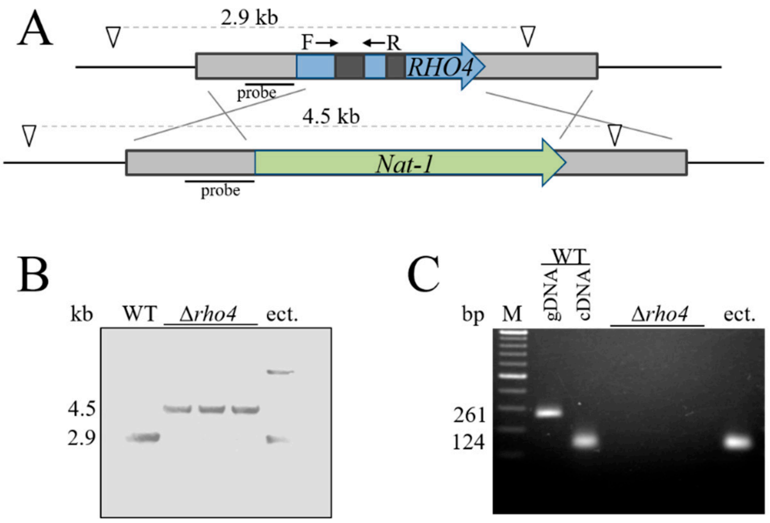

Figure S1. Deletion of *RHO4* of *C. graminicola* and identification of transformants with homologous and ectopic integration of the deletion cassette. **(A)** Deletion strategy of *RHO4* of *C. graminicola*. The gene *RHO4* is given in blue, introns are given in dark grey, 5′- and 3′-flanking regions in light grey. *Nat-1*, nourseothricin N-acetyl transferase gene. White arrowheads indicate *Xho*I restriction sites, black arrows labelled with F and R indicate positions of forward and reverse PCR primers. Black line below bars indicates position of the probe used in Southern blot analysis. **(B)** Southern blot analysis of the WT strain, the Δ*rho4* strains, and ectopic strains. **(C)** RT-PCR confirming the presence of *RHO4* transcripts in the WT strain and the ectopic strain, as well as the absence of *RHO4* transcripts in three randomly chosen Δ*rho4* strains. cDNA, complementary DNA; Δ*rho4*, deletion mutants; ect., ectopic strain; gDNA, genomic DNA; M, DNA size standard; WT, wildtype strain.

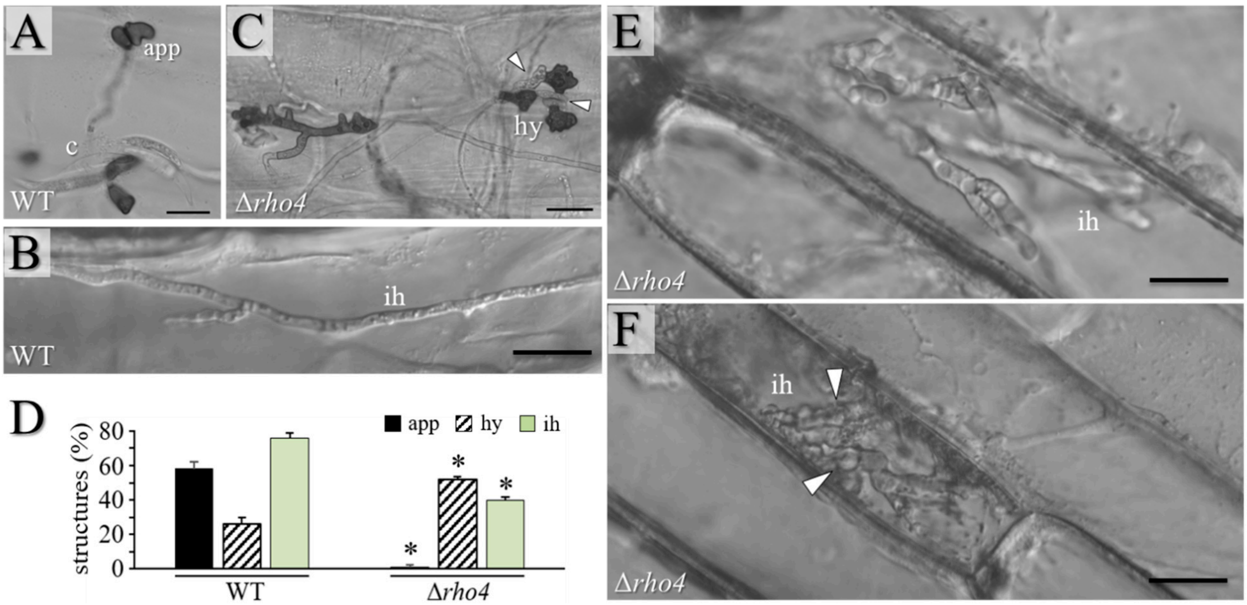

Figure. S2. Δ*rho4* mutants differentiate defective infection structures on the alternative host, *Allium cepa* cv. Shakespeare. **(A)** Conidia (c) of the *C. graminicola* WT strain germinate and differentiate in appressoria (app) and hyphopodia **(D)** on onion epidermal cells. **(B)** WT infection hypha (ih) formed after invading the onion epidermis. **(C)** The Δ*rho4* mutant differentiates hyphopodia (hy), which show lateral germination (arrowheads). **(D)** Appressorium, hyphopodium, and infection hypha differentiation by WT and Δ*rho4* strains. Error bars are standard deviations; asterisk indicates statistically significant differences of corresponding structures (T-test, $P \leq 0.05$). **(E** and **F)** In planta differentiated infection hyphae (ih) of the Δ*rho4* strain. Δ*rho4* infection hyphae densely colonize the host cell, with swellings indicative of cell wall defects (ih, arrowheads). Microscopy was done at 72 hpi. Size bars in (A – C, E and F) are 20 μm.
